# Concordance between estimates of acute malnutrition measured by weight-for-height and by mid-upper arm circumference after age adjustment: population-representative surveys from humanitarian settings

**DOI:** 10.1186/s40795-019-0301-z

**Published:** 2019-08-19

**Authors:** Eva Leidman, Alexia Couture, Erin Hulland, Oleg Bilukha

**Affiliations:** Emergency Response and Recovery Branch, Division of Global Health Protection, Center for Global Health, Centers for Disease Control, 1600 Clifton Road, Atlanta, GA 30329 USA

**Keywords:** Wasting, Survey, Nutrition, Humanitarian

## Abstract

**Background:**

Mid-upper arm circumference (MUAC) and weight-for-height (WHZ) are commonly used indicators to identify acute malnutrition. However, MUAC and WHZ diagnose different children, and produce prevalence estimates that are meaningfully different. Previous research in Somalia has suggested improved concordance using MUAC-for-age (MUACZ) rather than MUAC. We further evaluate the relationship between MUACZ, MUAC, and WHZ using surveys conducted globally.

**Methods:**

We analyzed 882 population representative surveys from 41 countries. Children ages 6–59 months were classified as acutely malnourished using three independent criteria: WHZ < − 2 (WHZ2), MUAC< 125 mm (MUAC125), MUACZ < − 2 (MUACZ2). Population prevalence using each of the three criteria are presented by country and region. Correlations of survey prevalence for each indicator pair were assessed. Multivariable regression models of MUACZ and MUAC125 adjusted for WHZ2, stunting prevalence, age, and sex. To evaluate individual level diagnostic concordance, we compared the proportion of children identified by each of the three criteria.

**Results:**

Median prevalence of acute malnutrition overall was highest for MUACZ2 (14.0%) followed by WHZ2 (10.6%), and lowest for MUAC125 (7.3%). The absolute difference in prevalence between MUACZ2 and WHZ2 was smaller than the difference between MUAC125 and WHZ2 for 51.3% of surveys. The correlations of WHZ2 with both MUACZ2 as well as with MUAC125 were weak, positive associations (Pearson’s r = 0.5757 and 0.4943, respectively), but MUAC125 and MUACZ2 had a strong, linear relationship (Pearson’s r = 0.9265). The adjusted regression model for MUACZ2 had greater fit (R^2^ = 0.50) relative to the adjusted model for MUAC125 (R^2^ = 0.43). The proportion of children identified by both MUAC125 and WHZ2 was 25.5%, smaller than the proportion identified by both MUACZ2 and WHZ2 (30.6%).

**Conclusions:**

MUACZ identified more children as malnourished than MUAC, resulting in a higher prevalence of acute malnutrition in nearly all settings. Prevalence by MUACZ was not consistently more similar to WHZ than that estimated by MUAC, and correlations with WHZ were only slightly improved relative to MUAC. Consequently, programmatic use of MUACZ cannot be justified based on improved concordance with WHZ. Further research on morbidity and mortality of children with low MUACZ only are needed before recommending MUACZ for wider use.

## Background

Presence of acute malnutrition in children is ascertained through anthropometric measurement. Two independent anthropometric indicators have been endorsed by the Standing Committee on Nutrition (SCN) and are widely used in determining admission into selective nutrition treatment programs as well as assessing population prevalence —weight-for-height Z-score (WHZ) and mid-upper arm circumference (MUAC) [1–3].

While these indicators intend to measure the same condition of acute malnutrition, numerous studies have found that the children identified for admission to treatment programs by the two indicators are not the same. A 2009 report by the World Health Organization (WHO) [3] noted that of all children identified as acutely malnourished by either indicator, only about 40% of children were identified by both. This finding of poor child level diagnostic concordance of these indicators has since been independently replicated using different samples of children. Based on analysis of data from 14,409 children in 4 countries, Roberfroid et al. [4] found that only 28.5% of children defined as acutely malnourished were diagnosed by both low MUAC and low WHZ. Similarly, Grellety et al. [5] reviewed data from 1,832 surveys containing measurements of 1,384,068 children from 47 countries, and found only 28.2% of children defined as acutely malnourished were diagnosed by both MUAC and WHZ. The proportion of children identified by both indicators in their study varied by country, but was consistently less than 40%.

Subsequent research has demonstrated that these diagnostic differences translated into meaningful variations in the population-prevalence of acute malnutrition determined by the two-indicators; a contrast to the initial intention of researchers to derive MUAC prevalence cut-offs for programmatic action and severity classification that corresponded with those produced by WHZ < -2 [6]. Based on analysis of 733 surveys from 41 countries, Bilukha and Leidman [7] found poor correlation between population prevalences of acute malnutrition by WHZ and by MUAC, even after adjusting for prevalence of stunting, sex and age ratios (R^2^ = 0.46). The research suggested that prevalence of WHZ was higher than MUAC in most surveys, but the reverse could be identified in select populations.

Researchers have hypothesized that MUAC-for-age (MUACZ) may have more diagnostic consistency with WHZ than MUAC due to the fact that MUACZ is adjusted by age and sex. As such, the calculation of the MUACZ indicator is similar to WHZ, both of which compare a child’s anthropometric measurements to an international reference population, whereas a single threshold is used for MUAC irrespective of age (125 mm) to classify acute malnutrition in children aged 6–59 months. Preliminary evidence from Somalia supports this hypothesis. Custodio et al. [8] found that prevalence of low MUACZ (15.8%) and WHZ (16.1%) from a pooled sample of 17 surveys conducted in Somalia between 2007 and 2016 were very similar, whereas prevalence of low MUAC (7.8%) was much lower. Analysis of children in the sample found that the proportion of acutely malnourished children identified by both WHZ and MUACZ (28.3%) was higher than the proportion of children identified by both WHZ and MUAC (18.1%).

Findings of improved diagnostic similarity of MUACZ and WHZ, relative to MUAC, may have broad practical implications. First, current global protocols for therapeutic feeding centers that recommend measuring mid-upper arm circumference nearly all include admission on low MUAC not low MUACZ. Second, measurement of MUAC using color-banded strips is widely used in humanitarian settings in large part given its simplicity, demonstrated to be feasible for low-literacy community members with limited training [9]. Screening children with MUACZ is more complex as it additionally requires both accurately ascertaining age and using a reference table to identify children with low MUACZ. Measuring age can be difficult and time-consuming in contexts with poor vital registration [9]. Use of the reference table requires both literacy and training [10]. Finally, as MUACZ is age standardized, use of the indicator would likely shift the mean age of children at admission. Absolute MUAC and age are correlated in the reference population; median MUAC values in the WHO standard population increase by approximately 17 mm between 12 and 59 months [8, 11]. Research by Hossain et al. [12], found that among Bangladeshi children a MUAC cutoff of 15 mm higher for children 37–60 months compared to children 6–24 months (140 v. 125 mm) had similar sensitivity and specificity as WHZ.

The aim of our study is therefore to further investigate the relationship between both indicator pairs (WHZ and MUAC v. WHZ and MUACZ) to assess differences in population prevalence and individual child diagnosis when using MUACZ vs. MUAC. Building on the work of Custodio et al. [8], using a larger and more diverse set of population representative surveys including children from countries globally, we aim to evaluate whether their findings hold for all regions. Given that MUACZ, but not MUAC, is an age standardized indicator, we also explore whether the relationship between MUACZ, MUAC and WHZ is consistent for all age groups. Further, to understand the practical implications of using MUACZ, we evaluate the diagnostic concordance of MUAC and MUACZ.

## Methods

Data reviewed for analysis were single stratum, cross-sectional, population representative surveys (subsequently referred to as “small-scale surveys”) provided by the United Nations High Commissioner for Refugees (UNHCR) and by Action Contre le Faim (ACF). Surveys were reviewed if they were conducted between 2013 and 2016 (UNHCR) and 2001–2017 (ACF) and measured age, sex, weight, height and MUAC in children aged 6–59 months. Survey datasets were examined for duplication prior to inclusion in the study.

Surveys with sample size smaller than 196 persons and cluster surveys with fewer than 25 clusters were excluded from all analyses as they did not meet minimum standards for small scale surveys [5, 13]. Surveys missing MUAC, height, weight, or cluster variable (if two-stage study design) for more than 20% of sampled children were also excluded from analysis. Additionally, surveys with standard deviations (SD) of mid-upper arm circumference (MUAC) greater than 160 mm or SD of the underlying z-score distribution greater than 1.3 for weight-for-height (WHZ) or MUAC-for-age (MUACZ) were excluded given concerns regarding quality of anthropometric measurements [14, 15]. Finally, a survey with an implausible ratio of males to females (> 125:1) was also excluded. Within surveys retained for analysis, children were excluded if age was missing or out of range (6.0–59.99 months) or if the child record was an exact duplicate of another for all variables including household and cluster number within the dataset.

Survey countries were categorized into seven regions: Latin America and the Caribbean; Eastern and Southern Africa; Democratic Republic of Congo (DRC); West and Central Africa; Southeast Asia and Pacific; Sudan; and Middle East and North Africa [16]. Sudan and DRC were kept as their own regions due to large number of surveys from each country.

Weight-for-height (WHZ), MUAC-for-age (MUACZ), and Height-for-age (HAZ) z-scores were calculated for all children using the WHO SAS macro, which applies the WHO 2006 growth standards [17]. Children with missing data for sex, weight, height or MUAC were excluded by indicator, such that a child with missing weight would be excluded from WHZ but not MUAC or MUACZ calculations. For each survey, prevalence of acute malnutrition was calculated independently for three indicators. Prevalence of acute malnutrition by WHZ (herein referred to as “WHZ2”) was defined as WHZ < -2. Prevalence of acute malnutrition by MUACZ (“MUACZ2”) was defined as MUACZ<-2. Prevalence of acute malnutrition by MUAC (“MUAC125”) was defined as MUAC< 125 mm. Independently for WHZ2 and MUACZ2, outlier observations were excluded from a survey if the Z-score of a child fell outside the flexible exclusion range of +/− 4 Z-scores from the observed survey sample mean, as described by WHO [18]. For MUACZ2, observations were excluded if they were less than 70 mm or greater than 220 mm. To describe the observed prevalences, we computed the medians and interquartile ranges (IQR) of survey level prevalence for each country, region, and overall (Table 1). Prevalences and summary statistics were produced for all children 6–59 months, as well as for two age categories of children, 6–23 months and 24–59 months.

**Table 1:** Prevalence of acute malnutrition among children 6–59 months by anthropometric indicator (WHZ, MUACZ and MUAC), by country and region

Region	Country	N Surveys	N children with WHZ	Median Prevalence WHZ < -2 (IQR)	N children with MUACZ	Median Prevalence MUACZ<-2 (IQR)	N children with MUAC	Median Prevalence MUAC< 125 (IQR)
Latin America & Caribbean	Bolivia	1	882	1.36	882	0.91	882	0.57
	Guatemala	2	1,420	1.98 (0.38–3.58)	1,429	7.20 (1.33–13.07)	1,430	4.52 (0.19–8.85)
	Haiti	24	13,268	4.49 (3.89–4.86)	13,272	6.28 (4.17–7.60)	13,306	3.16 (2.47–3.90)
	REGION TOTAL	27	15,570	4.35 (3.76–4.85)	15,583	6.25 (4.15–8.07)	15,618	2.99 (2.20–4.10)
Middle East & North Africa	Iraq	1	569	2.28 (2.28–2.28)	568	2.64 (2.64–2.64)	575	3.30 (3.30–3.30)
	Jordan	5	2,089	1.23 (0.92–1.44)	2,088	0.83 (0.62–0.96)	2,090	0.96 (0.41–1.06)
	Yemen	3	1,199	5.52 (3.58–16.93)	1,207	6.40 (2.05–9.66)	1,208	4.14 (2.38–6.53)
	REGION TOTAL	9	3,857	2.28 (1.23–3.58)	3,863	1.33 (0.83–2.64)	3,873	1.23 (0.96–3.30)
West & Central Africa	Burkina Faso	11	6,387	12.79 (7.54–16.09)	6,406	16.35 (9.18–18.75)	6,416	7.28 (3.61–10.32)
	Central African Republic	13	8,725	5.89 (5.56–7.72)	8,677	17.49 (14.7–20.76)	8,693	9.22 (7.61–9.73)
	Ivory Coast	1	699	4.29 (4.29–4.29)	700	0.57 (0.57–0.57)	700	0.14 (0.14–0.14)
	Cameroon	13	6,029	8.83 (6.90–11.93)	6,046	13.15 (8.81–14.70)	6,058	6.77 (4.49–7.62)
	Republic of Congo	58	52,545	7.88 (4.89–11.23)	52,488	15.31 (10.54–22.52)	52,557	7.90 (5.50–13.41)
	Guinea	5	4,034	6.44 (5.47–7.69)	4,047	7.52 (3.52–9.48)	4,055	4.01 (3.16–5.64)
	Liberia	6	1,961	3.41 (3.13–4.19)	1,977	4.81 (2.30–5.60)	1,978	2.61 (0.92–4.47)
	Mali	15	10,814	11.20 (8.70–16.14)	10,868	11.04 (5.43–15.95)	10,879	5.88 (2.62–7.65)
	Mauritania	6	3,671	12.68 (8.77–14.81)	3,687	9.94 (7.42–13.21)	3,692	5.46 (4.45–7.90)
	Niger	14	9,796	12.99 (11.40–15.77)	9,772	17.36 (14.94–24.31)	9,785	9.81 (7.25–16.18)
	Nigeria	2	969	22.03 (16.25–27.81)	976	25.03 (14.38–35.69)	980	18.51 (8.71–28.31)
	Sierra Leone	6	5,417	7.12 (6.43–7.40)	5,401	14.22 (11.15–15.78)	5,411	9.89 (7.67–10.67)
	Chad	67	38,245	9.76 (6.34–18.89)	38,233	7.62 (5.35–19.46)	38,266	3.76 (2.06–11.89)
	REGION TOTAL	217	149,292	9.46 (6.10–13.95)	149,278	13.00 (7.29–19.65)	149,470	6.69 (3.67–10.80)
Democratic Republic of Congo		105	91,026	8.58 (4.97–11.44)	90,983	16.70 (11.48–22.60)	91,150	10.15 (6.93–13.85)
Eastern & Southern Africa	Angola	1	869	5.98	866	10.85	869	5.29
	Burundi	7	3,484	5.73 (4.23–7.08)	3,496	7.08 (5.90–21.71)	3,499	3.03 (2.08–9.61)
	Djibouti	5	1,561	10.81 (10.36–13.84)	1,578	7.96 (7.85–11.89)	1,580	4.65 (3.78–5.75)
	Eritrea	2	671	20.22 (18.89–21.55)	674	8.63 (7.76–9.51)	674	4.31 (4.02–4.60)
	Ethiopia	55	26,144	16.91 (8.71–21.77)	25,961	11.29 (7.98–17.88)	25,991	5.09 (3.36–8.50)
	Kenya	55	34,039	11.11 (8.05–14.51)	34,148	9.62 (6.84–12.78)	34,173	3.76 (2.81–5.28)
	Madagascar	8	3,695	6.39 (3.49–7.64)	3,715	16.70 (11.69–20.74)	3,721	7.94 (5.48–10.65)
	Rwanda	12	4,439	4.71 (3.68–5.84)	4,439	4.28 (3.43–5.75)	4,441	2.28 (1.83–3.48)
	Somalia	4	3,549	15.55 (14.46–16.14)	3,556	20.86 (11.27–27.19)	3,558	11.27 (3.96–18.21)
	South Sudan	31	19,838	16.36 (9.31–25.16)	19,866	16.23 (10.26–18.66)	19,898	7.95 (5.60–9.52)
	Tanzania	5	2,476	1.90 (1.54–2.61)	2,484	5.69 (4.03–5.95)	2,485	1.18 (0.74–3.33)
	Uganda	35	34,006	6.02 (3.92–9.43)	34,014	12.41 (9.51–15.68)	34,068	7.60 (4.31–10.33)
	Zimbabwe	2	591	4.40 (4.38–4.42)	593	15.07 (3.36–26.78)	596	6.38 (2.35–10.40)
	REGION TOTAL	222	135,362	10.03 (6.20–16.87)	135,390	11.10 (7.48–16.19)	135,553	5.02 (3.11–8.24)
Sudan		136	117,640	18.60 (13.61–22.22)	117,471	17.52 (13.45–21.59)	117,593	9.55 (6.97–12.54)
East Asia & Pacific	Indonesia	3	1,599	20.10 (17.93–24.58)	1,599	22.18 (17.87–24.16)	1,599	9.24 (6.53–10.91)
	Myanmar	17	11,958	18.87 (8.49–20.82)	11,973	26.84 (23.74–30.18)	11,985	14.19 (10.58–15.06)
	Philippines	4	2,927	5.96 (4.74–8.67)	2,900	2.56 (1.93–3.51)	2,898	1.26 (0.95–1.62)
	REGION TOTAL	24	16,484	18.09 (6.15–20.46)	16,472	23.90 (13.93–28.20)	16,482	11.27 (5.00–14.98)
South Asia	Afghanistan	58	46,397	7.23 (5.50–9.37)	46,268	15.08 (12.14–23.14)	46,326	9.54 (6.90–14.26)
	Bangladesh	42	20,002	13.10 (11.69–15.54)	20,031	13.79 (8.96–17.61)	20,061	5.91 (4.54–7.89)
	India	8	3,597	23.76 (18.86–35.41)	3,604	27.10 (16.65–30.58)	3,604	11.19 (7.09–13.21)
	Nepal	12	6,874	12.21 (6.40–17.91)	6,885	19.83 (4.64–32.07)	6,889	8.87 (2.64–16.63)
	Pakistan	22	16,207	17.87 (11.83–20.85)	16,228	19.26 (17.34–29.20)	16,258	10.55 (8.49–17.07)
	REGION TOTAL	142	93,077	11.69 (6.83–15.54)	93,016	16.28 (11.43–23.14)	93,138	8.48 (5.60–13.10)
TOTAL		882	622,308	10.57 (6.28–16.51)	622,056	14.01 (8.81–19.95)	622,877	7.27 (4.39–11.19)

*MUAC* Mid-upper arm circumference, *MUACZ* Mid-upper arm circumference-for-age Z-score, *WHZ* Weight-for-height Z-Score, *IQR* Interquartile ranges

To examine the relationship between calculated prevalences for the three indicators, three analyses were performed. First, for each survey the difference in prevalence was calculated for each of the three indicator pairs (WHZ2 v. MUACZ2, WHZ2 v. MUAC125, and MUACZ2 v. MUAC125). Second, we plotted the correlations of survey level prevalences for each of the three indicator pairs. Both Spearman and Pearson’s correlations were calculated. Additionally, multivariable linear regression was modeled with MUACZ2 and MUAC125 prevalence as outcome. For each of the multivariable models, we included the following predictor variables, previously found to be associated with MUAC: WHZ2, stunting prevalence (HAZ < − 2), age ratio, and sex ratio of the survey sample. Age ratio was calculated as the proportion of children 6–23 months v. 24–59 months in the sample. All predictor variables were retained in the multivariable model regardless of significance in univariate models [4].

Finally, we examined diagnostic overlap of the indicators. For each of three pairwise comparisons (WHZ2 v. MUACZ2, WHZ2 v. MUAC125, and MUACZ2 v. MUAC125) we performed analysis on the subset of children that were diagnosed as acutely malnourished by both indicators or uniquely by one of the indicators in the pair. For these acutely malnourished children, by survey, the proportions of children in each of three categories were calculated: identified by both indicators in the pair, identified by only one indicator, or identified by only the other indicator. For this analysis, surveys were excluded if the number of acutely malnourished children in the sample was less than 10 children. Survey level analyses of diagnostic overlap are presented graphically.

All data aggregation, cleaning and analyses were performed using SAS version 9.3 [19] and all figures were produced using RStudio version 1.0.15 and the ggplot2 package [20, 21].

## Results

A total of 980 surveys conducted between 2001 and 2017 were reviewed. Of these, 39 surveys were excluded due to their design — small sample sizes (*n* = 20) or too few clusters (*n* = 19). Six surveys were excluded due to high proportion of missing values. Additionally, 53 surveys were excluded due to concerns about quality of the WHZ, MUAC or MUACZ measurements (*n* = 40), reported sex (*n* = 1), or within survey duplication (*n* = 12). For only diagnostic overlap, from the 882 final surveys, sixteen were excluded when less than 10 children were identified as malnourished by any criteria.

Of the 882 surveys retained for analysis, 207 surveys were obtained from UNHCR and 675 surveys were from ACF. Overall, 768 surveys used cluster design, 104 used a simple/systematic random sampling design, and 10 were exhaustive samples. Surveys median sample size was 709 children aged 6–59 months (IQR: 503–927). The majority of surveys were conducted in West and Central Africa (*n* = 217), Eastern and Southern Africa (*n* = 222), South Asia (*n* = 142) and Sudan (*n* = 136). The lowest number of surveys (*n* = 9) were available from the Middle East and North Africa (Table 1).

**Fig. 1:**
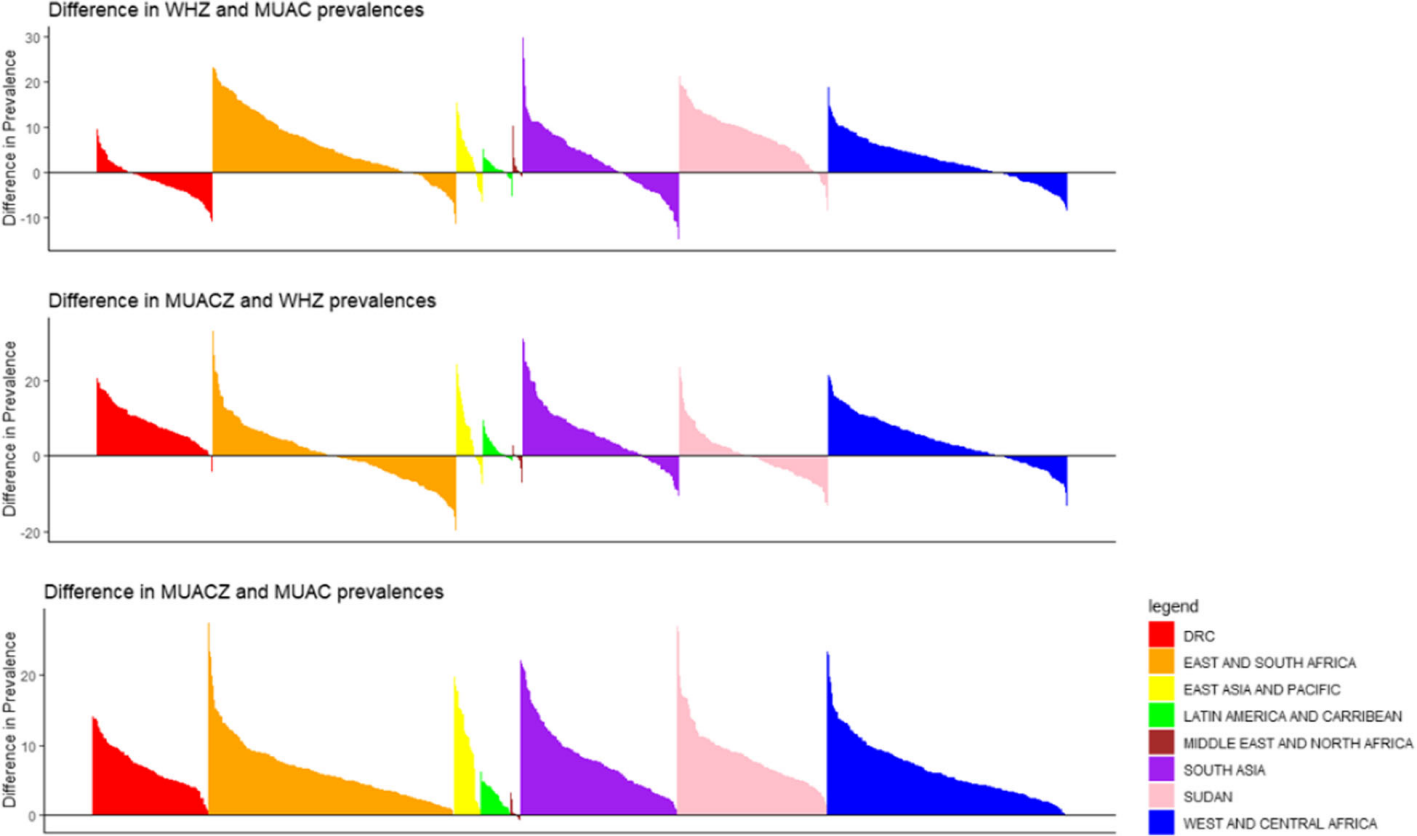
Survey-level differences in prevalence of acute malnutrition among children 6–59 months by anthropometric indicator pairs (WHZ and MUAC, MUACZ and WHZ, MUACZ and MUAC), by country (*n* = 882). Legend: Regions represented by colors are as follows: Democratic Republic of Congo (red), East and South Africa (orange), East Asia and Pacific (yellow), Latin America and Caribbean (green), Middle East and North Africa (brown), South Asia (purple), Sudan (pink), West and Central Africa (blue)

Table 1 presents the median prevalence of acute malnutrition for each of the three anthropometric indicators. Median prevalence of acute malnutrition overall was highest for MUACZ2 (14.0%) followed by WHZ2 (10.6%), and lowest for MUAC125 (7.3%). This held true in all regions except the Middle East and North Africa, where WHZ2 was higher than MUACZ2 (2.3 and 1.3%, respectively), and the DRC where MUAC125 was higher than WHZ2 (10.2 and 8.6%, respectively). Prevalence of WHZ2 was higher than MUAC125 in 70.3% of surveys and 76.2% of countries. Prevalence of MUACZ2 was higher than WHZ2 in 65.7% of surveys and 65.3% of countries. Prevalence of MUACZ2 was higher than MUAC in all but one survey (Table 1, Fig. 1). Figure 1 presents the difference in prevalence between each pair of indicators by survey, grouped by region, illustrating the range in magnitude of these differences. The comparisons of prevalence (Table 1, Fig. 1) also illustrate that overall, survey prevalence of WHZ2 was only marginally more similar to MUACZ2 than MUAC125, as the absolute difference between median MUACZ2 and WHZ2 (3.4%) is approximately the same as the absolute difference between median WHZ2 and MUAC125 (3.3%). The prevalence of WHZ2 was more similar to MUACZ2 than MUAC125 in 51.3% of surveys and 51.2% of countries.

**Fig. 2:**
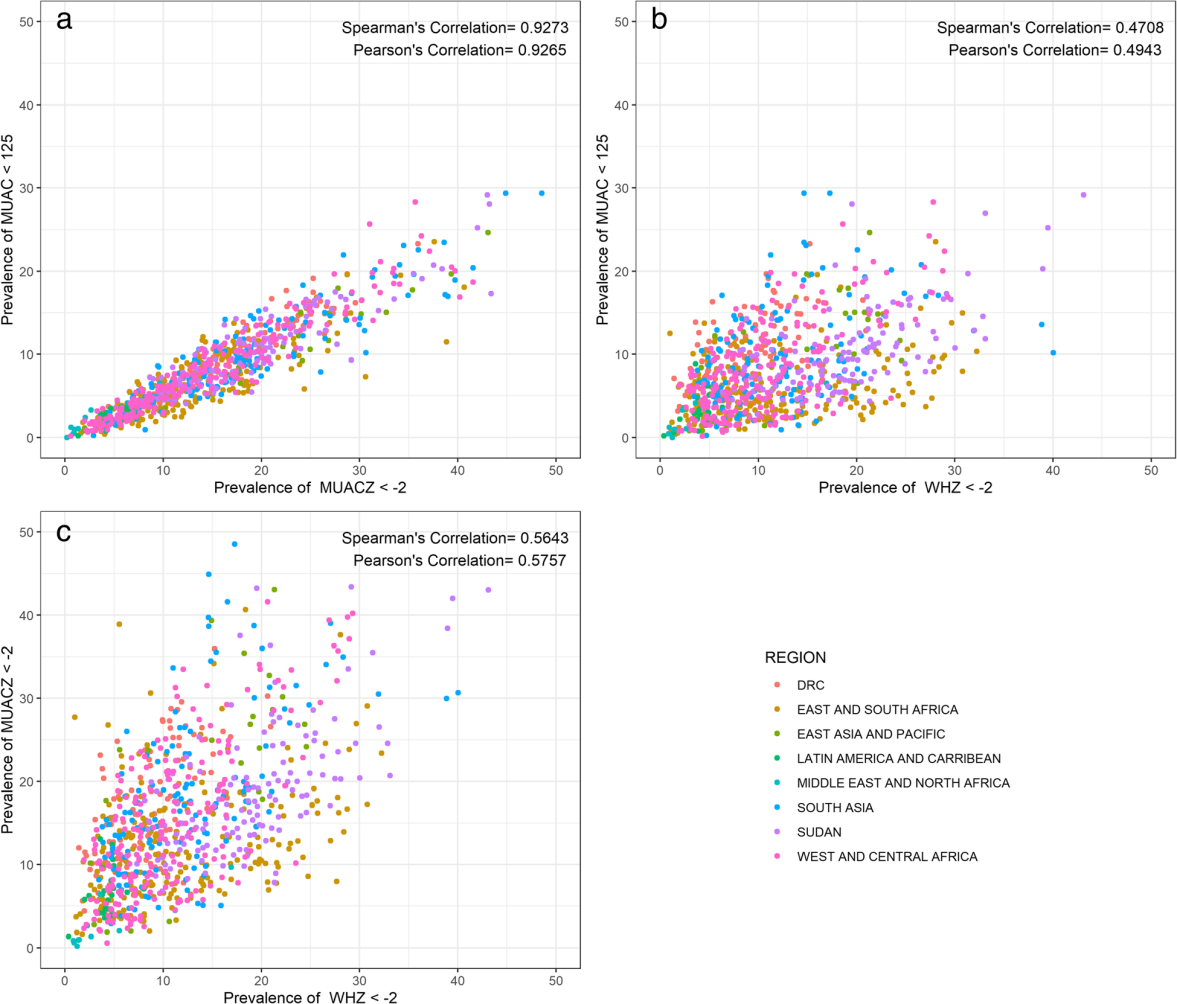
Pairwise correlation of prevalence of acute malnutrition indictor (MUAC and MUACZ, MUAC and WHZ, WHZ and MUACZ) (*n* = 882). Legend: Scatterplots of the correlation color coded by region between **a** prevalence of MUACZ < − 2 (x-axis) versus MUAC < 125 (y-axis), **b** prevalence of WHZ < − 2 (x-axis) versus MUAC < 125 (y-axis), **c** prevalence of WHZ < − 2 (x-axis) and prevalence of MUACZ < − 2 (y-axis). Regions represented by colors as follows: Democratic Republic of Congo (red), East and South Africa (dark yellow), East Asia and Pacific (green), Latin America and Caribbean (teal), Middle East and North Africa (turquoise), South (blue), Sudan (purple), West and Central Africa (pink)

Figure 2 presents the correlation between the prevalences for the three indicator pairs for each survey. The correlations of WHZ2 with both MUACZ2 and MUAC125 were weak, positive associations. The correlation between WHZ2 and MUACZ2 was slightly higher (Pearson’s r = 0.5757) than correlation between WHZ2 and MUAC125 (Pearson’s r = 0.4943). In contrast, the correlation between MUAC125 and MUACZ2 was a strong, linear, positive association (Pearson’s r = 0.9265). No clear regional patterns were observed.

Table 2 presents regression models with prevalence of MUACZ2 and MUAC as outcomes. Unadjusted models included only prevalence of WHZ2 as a predictor. Adjusted models additionally included HAZ2, age and sex ratios of survey samples as predictors. The model for MUACZ2 had higher fit (R^2^ = 0.50) relative to the unadjusted model where only WHZ2 was used as a predictor (R^2^ = 0.33). Similarly, the fit of the unadjusted model of MUAC125 v. WHZ2 improved after controlling for prevalence of stunting, age ratio, and sex ratio of the surveys (R^2^ = 0.24 and R^2^ = 0.43, respectively). For both adjusted and unadjusted models, R^2^ values were slightly higher for MUACZ2 than MUAC125. Coefficients for WHZ2 in both adjusted models were positive and less than 1.0, MUACZ2 (*β*_*WHZ*2_ = 0.84) and MUAC125 (*β*_*WHZ*2_ = 0.47). Coefficients of WHZ2 in the unadjusted models were smaller but not meaningfully different than those in the adjusted models [(*β*_*WHZ*2_ =0.70) and MUAC125 (*β*_*WHZ*2_ =0.36)]. Stunting was highly significant in both models (*p*-value < 0.0001), with small, positive coefficients (*β*_*HAZ*2_ = 0.23 for MUACZ2 and *β*_*HAZ*2_ = 0.14 for MUAC125). In the adjusted models, the age ratio of survey sample (6–23 months: 24–59 months) was not significant (p-value = 0.8796) in MUACZ2 model but highly significant (p-value < 0.0001) in the MUAC125 model. In the adjusted model for MUAC125, the age ratio variable had a strong, positive association with MUAC125 (*β*_*ageratio*_ = 7.15). The sex ratio was insignificant in both models.

**Table 2:** Multivariable regression models for prevalence of acute malnutrition diagnosed by MUACZ and MUAC

Survey-level analysis of prevalence	MUAC for Age		MUAC	
	Prevalence MUACZ < − 2		Prevalence MUAC < 125	
Variable	Estimate (95% CI)	*p*-value	Estimate (95% CI)	*p*-value
Acute malnutrition (WHZ < − 2) prevalence	0.84 (0.78–0.90)	< 0.0001	0.47 (0.43–0.50)	< 0.0001
Stunting (HAZ < -2) prevalence	0.23 (0.20–0.25)	< 0.0001	0.14 (0.12–0.15)	< 0.0001
Two-category age ratio (6–23 v. 24–59 months)	−0.29 (− 4.11–3.52)	0.8796	7.15 (4.70–9.60)	< 0.0001
Sex Ratio (Male to Female)	1.50 (− 3.28–6.28)	0.5389	0.56 (− 2.51–3.63)	0.7206
**Adjusted Model R**^**2**^	0.4968		0.4277	
Acute malnutrition (WHZ < − 2) prevalence	0.70 (0.63–0.76)	< 0.0001	0.36 (0.32–0.40)	< 0.0001
**Unadjusted Model R**^**2**^	0.3314		0.2444	

*MUAC* Mid-upper arm circumference, *MUACZ* Mid-upper arm circumference-for-age Z-score, *WHZ* Weight-for-height Z-Score, *HAZ* Height-for-age Z-Score

**Fig. 3:**
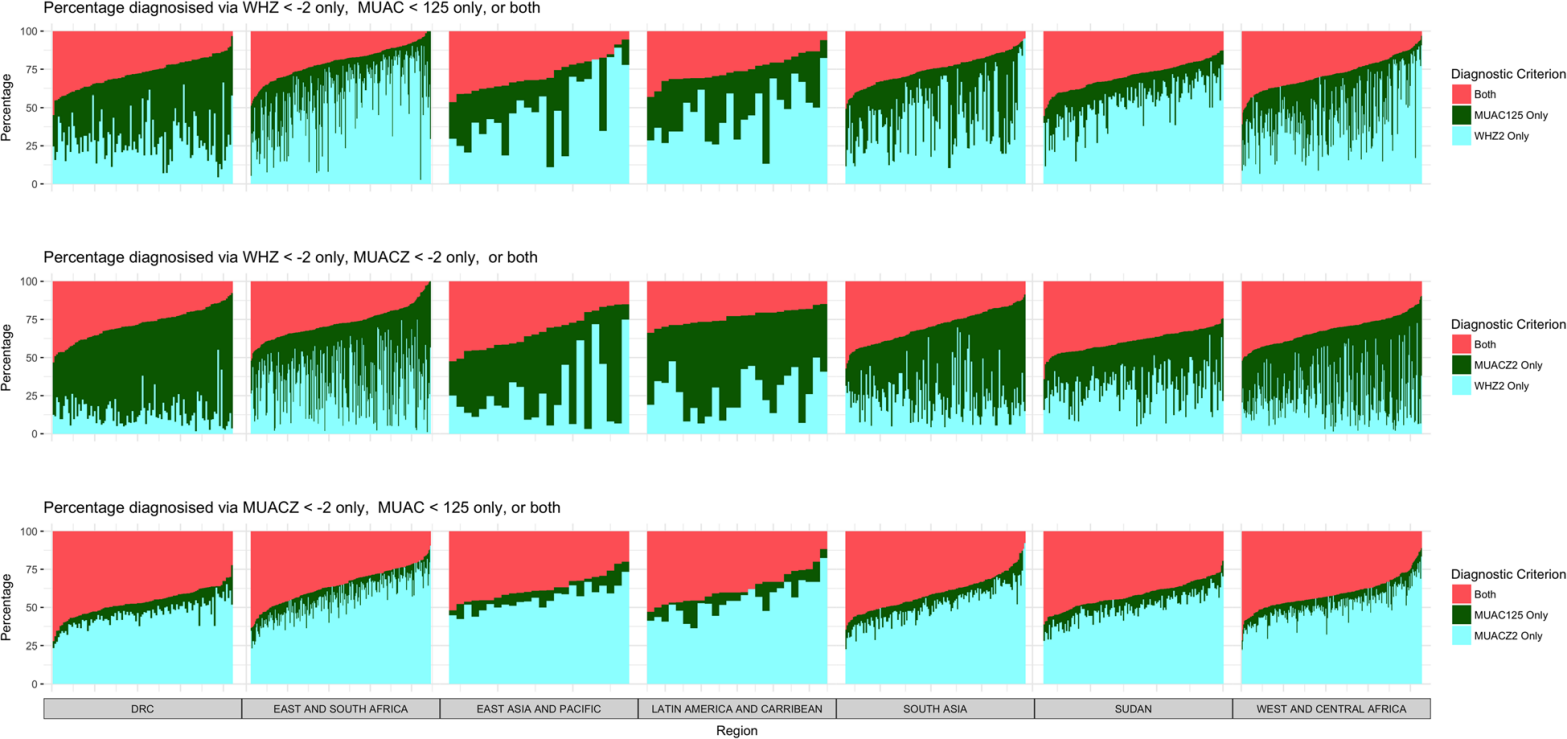
Contribution of each diagnostic criterion by survey, organized by region, for each of the three indicator combinations (WHZ and MUAC, MUACZ and WHZ, MUACZ and MUAC) (*n* = 863). Legend: Middle East region excluded given small number of surveys (*n* = 3) included in this analysis. Colors in the top row of graphs represent: both WHZ2 and MUAC125 (pink), MUAC125 only (green), WHZ2 only (blue). Colors in the middle row of graphs represent: both MUACZ2 and WHZ2 (pink), MUACZ2 only (green), WHZ2 only (blue). Colors in the bottom row of graphs represent: both MUAC125 and MUACZ2 (pink), MUAC125 only (green), MUACZ2 only (blue)

Analysis of diagnostic convergence of indicators for diagnosing acute malnutrition in individual children, presented in Table 3, found that overall the median proportion of children identified by both MUAC125 and WHZ2 was 25.5%. By region, the median proportion of children identified by both MUAC125 and WHZ2 was highest in East Asia and Pacific (32.0%) and lowest in Eastern and Southern Africa (17.7%). By survey, the proportion of acutely malnourished children diagnosed by both MUAC125 and WHZ2 ranged from 0 to 60.9% (Fig. 3). The median proportion of children identified by WHZ2 alone (48.2%) was higher than the median proportion of children identified by MUAC125 alone (21.6%). This held true across all regions except the DRC (42.1% MUAC125 only and 27.7% WHZ2 only).

**Table 3:** Proportion of acutely malnourished children 6–59 months of age diagnosed by each diagnostic indicator for three indicator pairs (MUACZ and WHZ, MUAC and MUACZ, MUAC and WHZ), by region^a^

Region	N surveys	MUAC < 125 and WHZ < -2				MUACZ <-2 and WHZ < -2				MUAC < 125 and MUACZ <-2			
		N children	Median Percentage MUAC only (IQR)	Median Percentage WHZ only (IQR)	Median Percentage of both (IQR)	N children	Median Percentage MUACZ only (IQR)	Median Percentage WHZ only (IQR)	Median Percentage of both (IQR)	N children	Median Percentage MUAC only (IQR)	Median Percentage MUACZ only (IQR)	Median Percentage of both (IQR)
Latin America & Caribbean	25	917	30.00 (18.18–34.33)	47.27 (34.33–59.09)	26.39 (20.69–30.91)	1,320	47.06 (37.50–60.00)	27.27 (16.95–38.10)	22.86 (20.00–27.12)	1,130	6.25 (5.13 - 11.28)	54.35 (47.62 - 62.07)	40.22 (33.33–45.45)
West & Central Africa	213	21,821	23.91 (12.00–37.75)	44.66 (32.20–62.39)	28.79 (20.63 - 35.29)	28,915	41.05 (30.57–55.28)	21.96 (12.92–35.48)	30.89 (25.85–39.36)	24,742	6.57 (4.92 - 9.09)	50.60 (45.04 - 57.58)	42.42 (35.82–46.77)
DRC	105	13,240	42.14 (33.02–54.29)	27.69 (21.00–37.57)	26.83 (20.00–34.16)	17,996	58.57 (48.85–65.98)	12.15 (8.43–19.08)	26.76 (20.13–35.24)	16,978	7.02 (5.22 - 9.09)	45.79 (41.29–50.86)	47.06 (40.88–52.11)
Eastern & Southern Africa	218	19,954	18.30 (9.17 - 32.47)	60.82 (44.83–74.36)	18.62 (11.94–26.47)	25,712	36.23 (20.19–53.57)	36.96 (19.05–48.72)	27.59 (20.69–33.65)	19,208	7.56 (4.46 - 11.11)	56.79 (46.15 - 66.67)	35.29 (26.13–43.71)
Sudan	136	26,423	15.30 (10.47–20.76)	54.93 (45.71–63.32)	28.26 (22.95–34.95)	31,672	28.44 (20.49–37.63)	31.19 (22.53–38.33)	38.84 (33.82–44.15)	24,440	7.89 (5.58 - 10.56)	50.00 (44.44–56.54)	42.18 (36.30–47.48)
East Asia & Pacific	24	3,124	20.32 (11.73–31.64)	46.64 (31.20–67.74)	31.97 (20.53–36.83)	4,407	40.11 (27.85–54.67)	18.14 (9.95–32.24)	33.98 (22.99–42.72)	3,872	6.43 (4.20 - 9.92)	54.08 (50.51 - 59.63)	38.96 (31.84–44.50)
South Asia	142	16,152	27.81 (15.18–45.99)	43.83 (27.15–60.18)	24.39 (18.07 - 31.68)	21,631	48.28 (31.02–61.8)	22.35 (14.98–31.53)	29.14 (21.03–38.60)	18,797	7.15 (5.49 - 10.34)	51.35 (43.01–59.33)	40.74 (33.33–49.08)
TOTAL^b^	866	101,766	21.64 (11.85–38.46)	48.19 (31.46–64.86)	25.53 (17.88–32.94)	131,794	41.38 (26.32–56.68)	24.09 (13.68–39.05)	30.63 (23.91–38.60)	109,267	7.04 (5.00 - 10.20)	51.01 (44.44–58.82)	40.78 (33.33–47.52)

*MUAC* Mid-upper arm circumference, *MUACZ* Mid-upper arm circumference-for-age Z-score, *WHZ* Weight-for-height Z-Score, *IQR* interquartile range, *DRC* Democratic Republic of Congo

^**a**^Median of the survey-level prevalence not pooled estimates; categories therefore do not add to 100%

^b^Total category includes 3 surveys from the Middle East that aren’t presented by regional breakdown due to small number of surveys

The overall median proportion of children identified by both MUACZ2 and WHZ2 was slightly higher (30.6%) than by both MUAC125 and WHZ2. By region, the median proportion of children in a survey identified by both MUACZ2 and WHZ2 was highest in Sudan (38.8%), and lowest in Latin America and Caribbean (22.9%). The proportion of acutely malnourished children diagnosed by both MUACZ2 and WHZ2 also varied considerably by survey (range: 0 to 64.2%) (Fig. 3). In contrast to the association of MUAC125 and WHZ2, the median proportion of children identified by WHZ2 alone (24.1%) was smaller than that of MUACZ2 alone (41.4%).

The median proportion of children identified by both MUAC125 and MUACZ2 in the overall analysis was higher than the diagnostic overlap of WHZ2 with either of those indicators, 40.8%. Median proportion of acutely malnourished children identified by both MUAC125 and MUACZ2 ranged from 35.3% in Eastern and Southern Africa to 47.1% in the DRC. The median proportion of children identified by MUACZ2 alone was 51.0% overall. The proportion of children identified by MUAC125 alone was 7.0% overall, and below 8.0% in all regions.

To further explore the finding that age ratio was significantly associated with MUAC125 but not MUACZ2 in multivariable models, median prevalences of all three indicators were stratified by age (6–23 months and 24–59 months), presented in Table 4. For WHZ2, prevalence of acute malnutrition among younger and older children was relatively similar (12.7 and 8.7%, respectively). The median difference in prevalence of WHZ2 between the two age groups was less than 10% in all regions. The prevalences of MUACZ2 were very similar for the two age groups, 13.9% for younger and 13.4% for older children. In contrast, the median prevalence of acute malnutrition by MUAC125 was more than five times higher for younger children (15.3%) relative to older children (2.8%). This difference was most pronounced in Sudan, where there was a difference of 18.6% between age groups’ median prevalences.

**Table 4:** Prevalences of global acute malnutrition produced by each indicator (WHZ, MUACZ and MUAC) by age group and by region

Region	Median Prevalence WHZ < − 2		Median Prevalence MUACZ<-2		Median Prevalence MUAC < 125 mm	
	Age 6–23 months [N] % (IQR)	Age 24–59 months [N] % (IQR)	Age 6–23 months [N] % (IQR)	Age 24–59 months [N] % (IQR)	Age 6–23 months [N] % (IQR)	Age 24–59 months [N] % (IQR)
Latin America & Caribbean	[5,830] 5.65 (4.18–6.96)	[9,740] 3.21 (2.09–3.89)	[5,837] 4.88 (3.76–8.40)	[9,746] 6.29 (3.92–8.93)	[5,852] 5.51 (3.74–8.92)	[9,766] 1.48 (0.77–2.38)
Middle East & North Africa	[1,400] 2.11 (0.88–4.76)	[2,457] 1.60 (1.11–3.14)	[1,409] 3.45 (0.88–5.70)	[2,454] 0.95 (0.37–1.09)	[1,410] 2.76 (2.63–6.22)	[2,463] 0.47 (0.00–0.94)
West & Central Africa	[53,008] 12.10 (7.79–18.28)	[96,284] 7.42 (4.36–11.44)	[52,882] 12.75 (7.59–19.46)	[96,396] 12.45 (7.00–20.41)	[52,977] 13.96 (7.95–21.27)	[96,493] 2.75 (0.96–5.69)
Democratic Republic of Congo	[34,280] 9.42 (5.76–13.81)	[56,746] 6.81 (4.37–10.30)	[34,232] 15.86 (12.07–21.99)	[56,751] 16.31 (11.78–23.08)	[34,319] 17.84 (13.22–24.08)	[56,831] 5.01 (2.79–7.31)
Eastern & Southern Africa	[49,039] 10.60 (7.91–16.30)	[86,323] 9.36 (4.76–17.18)	[48,961] 9.77 (5.84–14.71)	[86,429] 11.43 (7.43–16.84)	[49,029] 10.76 (6.31–16.22)	[86,524] 1.76 (0.74–3.40)
Sudan	[39,888] 22.84 (16.76–29.68)	[77,752] 16.22 (10.52–19.38)	[39,607] 19.98 (14.26–25.91)	[77,864] 16.44 (12.06–20.68)	[39,685] 21.42 (16.36–29.41)	[77,908] 2.85 (1.80–5.12)
East Asia & Pacific	[5,757] 22.49 (10.58–27.10)	[10,727] 13.83 (4.18–16.72)	[5,728] 20.96 (10.91–26.57)	[10,744] 25.90 (14.14–29.72)	[5,733] 22.08 (9.72–30.67)	[10,749] 4.56 (1.61–6.90)
South Asia	[32,422] 14.82 (8.97–21.74)	[60,655] 8.48 (5.15–13.95)	[32,226] 17.49 (11.38–25.26)	[60,790] 15.18 (10.18–21.12)	[32,313] 18.47 (12.55–27.29)	[60,825] 3.06 (1.68–5.19)
TOTAL	[221,624] 12.72 (8.17–20.67)	[400,684] 8.73 (4.80–14.74)	[220,882] 13.92 (8.56–20.22)	[40,1174] 13.41 (8.56–20.22)	[221,318] 15.32 (9.17–22.80)	[401,559] 2.75 (1.31–5.01)

*MUAC* Mid-upper arm circumference, *MUACZ* Mid-upper arm circumference-for-age Z-score, *WHZ* Weight-for-height Z-Score, *IQR* interquartile range

Consequently, the pattern of diagnostic overlap of the indicators was different for younger and older children (Table 5). For MUACZ2 and WHZ2, the proportion of children identified by both was similar for younger and older groups (35.8% versus 28.0%). Whereas, for MUAC125 and WHZ2, the proportion of children identified by both was much higher for younger children (34.8%) than older children (14.0%). Notably, among older children, the proportion of children identified by both MUACZ2 and WHZ2 was nearly double the proportion identified by both MUAC125 and WHZ2; by contrast, in younger children the proportions were approximately equal (35.8% v. 34.8% respectively). The proportion of children diagnosed by MUAC125 and MUACZ2 were very different in younger and older children (70.5 and 20.0%, respectively). Among older children, all acutely malnourished children not identified by both indicators were diagnosed only by MUACZ2; MUAC125 had no added diagnostic benefit.

**Table 5:** Proportion (IQR) of acutely malnourished children 6–23 and 24–59 months of age diagnosed by each of three indicator pairs (MUACZ and WHZ, MUAC and MUACZ, MUAC and WHZ), by region^a^

	Children 6 to 23 Months of Age				Children 24 to 59 Months of Age			
Region	N Children	Median Percentage MUACZ only (IQR)	Median Percentage WHZ only (IQR)	Median Percentage of both (IQR)	N children	Median Percentage MUACZ only (IQR)	Median Percentage WHZ only (IQR)	Median Percentage of both (IQR)
Latin America & Caribbean	491	33.33 (21.43–38.46)	33.33 (20.00–50.00)	33.33 (29.41–41.38)	829	62.32 (53.33–72.86)	15.71 (11.76–28.57)	16.13 (11.43–23.19)
West & Central Africa	10,873	28.89 (16.67–46.81)	28.89 (17.24–46.67)	36.84 (28.57–43.75)	18,042	51.79 (38.46–64.75)	16.07 (10.00–30.00)	28.71 (22.22–36.62)
DRC	6,875	53.70 (38.18–62.50)	14.29 (8.70–23.21)	30.43 (23.81–38.98)	11,121	63.79 (53.95–71.57)	10.20 (7.08–15.63)	24.60 (17.50–30.67)
Eastern & Southern Africa	8,907	26.67 (15.79–41.03)	41.15 (25.93–54.55)	31.40 (20.83–39.53)	16,805	43.39 (21.98–65.45)	31.91 (14.10–46.97)	25.00 (16.67–32.50)
Sudan	12,016	20.23 (14.09–31.56)	32.11 (23.80–40.00)	45.82 (38.31–51.39)	19,656	33.00 (24.59–45.26)	27.86 (21.72–38.27)	34.72 (30.04–39.78)
East Asia & Pacific	1,564	25.21 (15.63–38.56)	25.58 (18.34–49.48)	37.22 (27.91–47.54)	2,843	49.05 (36.18–66.87)	12.74 (6.19–21.46)	29.59 (17.49–44.38)
South Asia	8,558	33.58 (18.52–50.98)	27.64 (17.24–42.86)	33.69 (24.73–43.86)	13,073	55.46 (37.50–70.64)	18.80 (9.76–27.27)	24.66 (17.07–34.51)
TOTAL^b^	49,350	30.37 (17324–45.76)	30.36 (16.95–45.61)	35.78 (26.48–44.44)	82,444	49.06 (31.58–65.88)	19.96 (10.28–35.09)	28.00 (19.35–35.53)
Region	N Children	Median Percentage MUAC only (IQR)	Median Percentage MUACZ only (IQR)	Median Percentage of both (IQR)	N children	Median Percentage MUAC only (IQR)	Median Percentage MUACZ only (IQR)	Median Percentage of both (IQR)
Latin America & Caribbean	442	19.23 (14.29–25.00)	16.00 (13.43–20.00)	64.18 (54.55–71.43)	688	0	76.67 (73.68–83.33)	23.33 (16.67–26.32)
West & Central Africa	9,686	16.67 (13.04–22.50)	11.11 (7.14–15.79)	71.67 (64.29–76.34)	14,266	0	77.78 (70.97–85.71)	22.22 (14.29–29.03)
DRC	7,088	17.78 (13.64–21.43)	10.39 (7.02–13.21)	72.22 (65.63–77.02)	9,890	0	72.97 (64.14–79.63)	27.03 (20.37–35.86)
Eastern & Southern Africa	7,232	20.00 (14.29–27.27)	11.76 (7.14–18.18)	66.67 (57.58–74.03)	11,976	0	83.89 (77.95–90.00)	16.11 (10.00–22.05)
Sudan	10,174	18.84 (13.68–23.61)	9.52 (6.62–12.89)	71.43 (66.26–76.30)	14,266	0	80.90 (73.97–86.22)	19.10 (13.78–26.03)
East Asia & Pacific	1,366	17.70 (13.03–21.83)	10.66 (5.41–17.03)	72.54 (66.67–77.50)	2,506	0	81.37 (75.42–87.39)	18.63 (12.61–24.58)
South Asia	7,876	18.37 (13.25–23.88)	9.82 (7.14–14.29)	71.06 (63.89–77.27)	10,921	0	79.41 (72.34–86.11)	19.10 (13.78–26.03)
TOTAL^b^	43,920	18.35 (13.64–24.32)	10.71 (7.04–15.09)	70.50 (63.08–76.19)	65,347	0	80.00 (72.73–86.36)	20.00 (13.64–27.27)
Region	N Children	Median Percentage MUAC only (IQR)	Median Percentage WHZ only (IQR)	Median Percentage of both (IQR)	N children	Median Percentage MUAC only (IQR)	Median Percentage WHZ only (IQR)	Median Percentage of both (IQR)
Latin America & Caribbean	522	35.29 (26.32–38.46)	28.13 (21.43–50.00)	32.35 (23.08–41.67)	395	16.67 (5.88–32.26)	57.14 (47.62–73.91)	21.43 (14.29–25.00)
West & Central Africa	11,437	33.33 (17.39–48.48)	25.76 (14.71–43.48)	35.19 (27.03–44.44)	10,384	13.16 (3.45–25.61)	64.29 (47.80–82.81)	17.39 (10.00–26.92)
DRC	7,456	56.82 (41.18–67.27)	13.33 (7.84–19.67)	28.72 (21.95–38.17)	5,784	24.32 (15.15–36.36)	49.21 (36.17–58.82)	25.58 (14.29–30.99)
Eastern & Southern Africa	9,416	30.00 (19.29–44.44)	35.76 (24.15–50.55)	30.63 (22.50–38.64)	10,538	6.49 (0.86–20.00)	85.04 (61.29–93.33)	7.69 (2.61–15.38)
Sudan	12,696	25.00 (18.02–33.03)	29.24 (20.47–37.28)	45.63 (37.46–51.46)	13,727	4.95 (2.78–10.22)	81.90 (69.00–88.29)	12.43 (8.67–19.33)
East Asia & Pacific	1,610	30.35 (19.30–38.49)	21.90 (17.34–47.93)	41.49 (30.75–46.85)	1,514	9.55 (3.34–27.92)	70.71 (48.98–84.41)	18.91 (8.45–27.21)
South Asia	9,181	35.86 (24.62–53.85)	24.05 (14.29–39.29)	32.33 (23.81–43.20)	6,971	13.25 (4.00–31.58)	68.99 (47.62–82.05)	15.38 (9.80–21.74)
TOTAL^b^	52,391	33.33 (20.50–48.28)	26.92 (15.38–27.27)	34.78 (25.49–44.19)	49,375	11.11 (2.90–25.00)	70.75 (50.00–86.68)	14.03 (7.41–24.53)

*MUAC* Mid-upper arm circumference, *MUACZ* Mid-upper arm circumference-for-age Z-score, *WHZ* Weight-for-height Z-Score, *IQR* interquartile range

^**a**^Median of the survey-level percentage not pooled estimates; categories therefore do not add to 100%

^b^Total category includes 3 surveys from the Middle East that aren’t presented by regional breakdown due to small number of surveys

## Discussion

The primary aim of our analysis was to evaluate the finding by Custudio et al. [8] that acute malnutrition prevalence as measured by MUACZ more closely approximated that of WHZ than absolute MUAC. This finding was also previously supported by a study of Bangladeshi children; a cross sectional survey of 27,767 children – which found that cutoffs for MUAC differed according to age groups when looking at acute malnutrition obtained with WHZ2, suggesting MUACZ as an age-adjusted indicator could resolve those differences [12]. Our analysis, the first multi-country analysis to explore this association, demonstrates that the improvement in fit overall is minor and does not hold for all countries or regions. Overall, the difference in median prevalence comparing WHZ2 and MUACZ2 was approximately the same as the difference in median prevalence comparing WHZ2 and MUAC125, about 3%. WHZ2 was more similar to MUAC125 in half of the surveys and more similar to MUACZ2 in the other half. In the majority of surveys, WHZ2 was greater than MUAC125, and MUACZ2 was greater than WHZ2. However, there were surveys and countries where the reverse relationships were found. The correlation of MUACZ2 and WHZ2 (Pearson’s r = 0.5757) was slightly higher than the correlation of MUAC125 and WHZ2 (Pearson’s r = 0.4943), but both indicators were poorly correlated with WHZ. Both associations were somewhat improved after controlling for stunting, sex and age ratios in regression models.

In addition to population level associations, we examined child level diagnostic implication of using MUACZ, MUAC and WHZ. Custudio et al. [8] previously found that the proportion of children identified as acutely malnourished by both WHZ2 and MUACZ2 was higher than the proportion identified with both WHZ2 and MUAC125. Our analysis confirmed this finding; overall, overlap for WHZ2 and MUACZ2 was slightly higher (30% of malnourished children) than WHZ2 and MUAC125 (25%). However, the proportion of children identified by each indicator, and thus the relative improvement, varied considerably by region. The proportion of acutely malnourished children identified by WHZ2 as well as MUAC125 was lower than by WHZ2 and MUACZ2 in nearly all regions. However, in Latin America and the Caribbean, the reverse was true; the overlap of WHZ2 was greater for MUAC125 (26.4%) than MUACZ2 (22.9%). Our analysis documents considerable variability by survey within regions, and even within countries. Previous research has documented increased mortality risk among children with both WHZ2 and MUAC125 relative to those identified as malnourished by only one indicator; the relative risk of children with both WHZ2 and MUACZ2 has not been investigated [22]. Among malnourished children not identified by both indicators, MUACZ2 diagnosed more children as malnourished than WHZ2. This is a contrast to the relationship between WHZ and MUAC, in which more children have low WHZ but not low MUAC than the reverse. However, these proportions also varied notably by survey, country and region.

The major factor that distinguishes absolute MUAC and MUACZ is the age-standardization. Consistent with expectations and previous research [4, 11], in our multivariable models, age ratio was associated with prevalence of MUAC125 only. The median prevalence of acute malnutrition by MUACZ2 was similar in younger and older children, but five times higher in younger children than in older for MUAC125. Consequently, we were interested in further exploring the impact of age on the association between WHZ and the two MUAC indicators. The proportion of malnourished children identified by WHZ2 as well as MUACZ2 were similar in the younger (6–23 months) and older (24–59 months) age groups (35.8% v. 28.0% v, respectively). Whereas, the proportion of malnourished children identified as both WHZ2 and MUAC125 was nearly double for younger children (34.8% v. 14.0%, respectively). These data suggest a similar relationship between WHZ2 and both MUAC indicators among younger children, but greater discordance for MUAC125 than MUACZ2 for older children.

Since the development and publication of the MUAC-for-age reference data in 1997 [11], there has been limited research exploring the association between MUAC and MUACZ. We know of only one small study in Nigeria that directly calculated the diagnostic overlap of the two indicators and found that 35.3% of acutely malnourished children 6–59 months measured were diagnosed by both MUACZ2 and MUAC125; 17.7% were MUACZ2 only and 47.0% were MUAC125 only [23]. Notably, the sample had a disproportionately large number of younger children, 60.8% of the children were 6–23 months of age. In contrast, our study found that overall, MUACZ2 identified more children than MUAC125. The median prevalence of MUACZ2 in nearly all surveys was notably higher than MUAC125; this finding was very consistent across regions. Unlike the weak association between WHZ2 and both MUAC indicators, the correlation between MUACZ2 and MUAC125 was high (R^2^ ≈ 0.85). Consequently, it may be possible to devise a relatively reliable formula for conversion of MUAC125 into MUACZ2 prevalence, as was done previously for converting estimates from National Center for Health Statistics (NCHS) growth reference to estimates using the WHO growth standards where a high degree of fit (R^2^ > 0.9) was observed [24].

Interestingly, while the association between MUACZ2 and MUAC125 was strong, only 40% of children were identified by both indicators. The proportion of children identified by both is very age dependent. Both MUAC125 and MUACZ2 identified similar children in the younger age group (70% overlap). However, in the older age group, only 20% of malnourished children were identified by both indicators. The finding that MUAC125 identified no children aged 24 months or older not otherwise identified by MUACZ2, conforms with expectations but has not been described previously in the research. The programmatic implications of this require more research. Data on the prognostic ability of MUACZ2 to predict mortality, especially in older age group, is limited. A study in Guinea-Bissau found no significant difference comparing MUAC125 and MUACZ2 in ability to predict mortality at 30 and at 90 days among children aged 6–35 months [25]. Studies including a broader age group, a longer follow-up period, and multi-country comparisons are not available. Recent study developing and testing MUAC for age growth reference for children aged 5–17 years suggested that MUAC for age is at least as effective as body mass index for age for assessing mortality risks among African school aged children [26].

This study is subject to several limitations. First, analysis is limited to sub-national surveys conducted primarily in emergency settings that were made available for review. These subnational surveys are conducted more frequently in countries with poor nutritional status and ongoing nutritional programming. Consequently, few surveys from Latin American countries and no European countries were available for analysis. In addition, large multi-indicator national surveys, such as Demographic and Health Surveys, measure WHZ but not MUAC or MUACZ. While the final dataset included surveys from 41 countries, the geographic distribution may impact generalizability of findings. Additionally, the surveys included in our study were designed to achieve reasonable precision for global acute malnutrition. The number of children with severe acute malnutrition within these samples was consequently quite small. Therefore, future analysis looking at the association of severe rather than total acute malnutrition by WHZ, MUAC and MUACZ, would require surveys with larger samples or a pooled analysis design. Finally, analysis of age differences requires accurate age estimation. Settings with high acute malnutrition also tend to have poor vital registration. Age is consequently estimated to the nearest month for many of the children (where exact date of birth is not available). Imperfect age estimation may impact the associations described.

## Conclusions

The presented analysis demonstrates some limited improvement in convergence with WHZ when using MUACZ instead of MUAC. Wider use of MUACZ for programming, however, cannot be justified based on these limited improvements. In terms of field logistics, MUAC is more convenient and quick to measure, as MUACZ additionally requires age estimation and use of field-reference tables. Additionally, limited research exists on whether children with low MUACZ have an increased risk of mortality, as has been previously documented for low MUAC and low WHZ. Finally, use of MUACZ for admission to treatment should be informed by research on responsiveness of children with low MUACZ, but not low MUAC or low WHZ, to nutrition treatment.

## Data Availability

The data that support the findings of this study are available from Action Contre la Faim and the United Nations High Commissioner for Human Rights but restrictions apply to the availability of these data, which were used under license for the current study, and so are not publicly available. Data are however available from the authors upon reasonable request and with permission of Action Contre la Faim and the United Nations High Commissioner for Human Rights.

## Abbreviations

ACF: Action Contre le Faim
DRC: Democratic Republic of Congo
GAM: Global acute malnutrition
HAZ: Height-for-age
IQR: Interquartile ranges
MUAC: Mid-upper arm circumference
MUAC125: MUAC < 125 mm
MUACZ: MUAC-for-age
MUACZ2: MUACZ < − 2
SAM: Severe acute malnutrition
SCN: Standing Committee on Nutrition
SD: Standard deviations
UNHCR: United Nations High Commissioner for Refugees
WHO: World Health Organization
WHZ: Weight-for-height z-score
WHZ2: WHZ < − 2
